# Coping strategies and their associated factors among caregivers of patients with schizophrenia in Kuantan, Malaysia

**DOI:** 10.3389/fpsyt.2022.1004034

**Published:** 2022-09-29

**Authors:** Tengku Mohd Saifuddin Tengku Kamarulbahri, Suthahar Ariaratnam, Azlina Wati Nikmat, Nik Nairan Abdullah, Tan Lee Khing

**Affiliations:** ^1^Department of Psychiatry, Faculty of Medicine, Universiti Teknologi MARA (UiTM), Sungai Buloh, Malaysia; ^2^Department of Public Health Medicine, Faculty of Medicine, Universiti Teknologi MARA (UiTM), Sungai Buloh, Malaysia; ^3^Psychiatric Department, Tengku Ampuan Afzan Hospital, Kuantan, Malaysia

**Keywords:** coping strategies, caretaker, psychological distress, schizophrenia, stress

## Abstract

**Background:**

It is essential for caregivers of schizophrenia patients to have effective coping mechanisms to deal with their own mental health. However, research on the factors that contribute to the coping mechanisms of caregivers is limited. The purpose of this study is to investigate the use of coping strategies and their correlations to socio-demographic features, psychological distress, and social support among the caregivers of patients with schizophrenia.

**Method:**

Through the convenience sampling method, 331 caregivers of patients with schizophrenia participated in this cross-sectional study. The respondents comprised caregivers who had attended an outpatient psychiatric clinic, providing the socio-demographic data. The study involves three instruments: the Kessler's Psychological Distress (K10); the Multidimensional Scale of Perceived Social Support (MSPSS); and the Brief-COPE.

**Findings:**

Using multivariable analysis, psychological distress was identified as a substantial independent predictor of emotion-focused, problem-focused, and avoidance coping strategies. Good social support was linked to the usage of problem-focused and emotion-focused coping strategies.

**Conclusion:**

It has been demonstrated that good social support and psychological distress are associated with coping strategies. More prospective and qualitative research is required to determine how coping strategies will be able to assist Malaysian caregivers to develop a more holistic approach to treating patients with schizophrenia.

## Introduction

Schizophrenia is a mental illness that has a substantial negative impact on a patient's social life. It also results in substantial personal distress. In addition to the fact that hospitals no longer have to shoulder the burden of care, caregivers and society also suffer significant immediate and long-term consequences, such as long-term psychosocial and economic support, frequent hospitalization, and work productivity loss throughout the patients' lifetime (1). In most cases, schizophrenia patients show signs of social impairment, which is a major cause of distress to caregivers and patients (2). The emotional well-being of caregivers and the ability to lead a fulfilling personal and family life have all been reportedly affected by the illness (1). Besides, family caregivers experience stress and exhaustion due to the demanding task of providing care for schizophrenia patients over an extended period of time (3).

Subsequently, it is important to identify appropriate coping styles to reduce the burden on caregivers of patients with schizophrenia. However, it is difficult to determine the coping strategies and their associated factors used by the caregivers, which may pose challenges and obstacles to the care of schizophrenia patients. Despite the critical component of providing support to schizophrenia patients' caregivers and improving their coping strategies in the context of good clinical practice (4), caregivers still experience psychological distress. In Malaysia, 31.5% of schizophrenia patients' caregivers have been reported to suffer psychological distress at some point in life (2). Therefore, to ease the caregivers' distress, the healthcare provider should promote adaptive coping strategies as opposed to maladaptive strategies (2). Moreover, the use of regular adaptive coping strategies among caregivers has been linked to better functionality among patients (5).

Coping strategies encompass behavioral and cognitive efforts to reduce, handle, or endure internal or external demands (4). Coping strategies are also employed to alleviate the consequences (4). The caregivers of patients with schizophrenia employ both effective and ineffective coping strategies to cope with their difficulties (6). The coping strategies varied among them (7). The different coping styles were reclassified into two groups: problem-focused and emotion-focused (8). A problem-focused coping approach has been traditionally linked to better physical and mental health as well as a reduction in caregiver burden, resulting in improved patient coping (9). In terms of emotional focus, individuals who interpret the circumstances in a different way without changing the actual person-environment relationship, such as people who use resignation, coercion, or avoidance, rather than altering the person-environment relationship, are thought to suffer more and have a higher relapse rate when dealing with stressful situations (10).

Lazarus and Folkman reported that effective coping strategies depend on the situation. In some cases, the use of both coping strategies might offer the best solution (11). The emotion-focused coping strategy is the best for stressors over which the sufferer does not have control, such as the loss of a loved one or the diagnosis of a terminal illness (12). Manageable situations necessitate more problem-focused coping strategies since the person can change them (11). A brief reduction in stress can be achieved by exposing feelings as a coping mechanism, but it often increases the risk of future issues, including depression and neglect of the patient being cared for (13). How long the stressor lasts must be considered while determining effectiveness. Long-term stressors require more focused attention, while short-term stressors prefer avoidance strategies (11). Apart from emotion-focused coping and problem-focused coping, sometimes there is a third category called avoidance coping, which includes self-blame, substance use, denial, venting, behavioral disengagement, and self-distraction (14). The avoidance coping strategies are linked to the functional impairment of the patient (15).

Thus, their coping strategies should be explored to provide insights into how they are coping when facing the illness. Moreover, developing an intervention for coping strategies is necessary to reduce the stress level of the caregiver of a patient with schizophrenia that arises from various related stressors. Understanding their coping strategies is essential to improving the health-related outcomes of patients with schizophrenia. Therefore, for a better understanding of coping strategies, this research aims to determine their use and association with the sociodemographic characteristics, social support, and psychological distress among the caregivers of patients with schizophrenia. The research on coping strategies among caregivers of patients with schizophrenia mainly focused on their association with psychological distress (5), caregiver burden (16), maladaptive schemas (17), patient psychopathology (8), disease perceptions (18) and sociodemographic variables of patients (16). To the best of our knowledge, there is a paucity of local studies looking into the association between perceived social support and psychological distress with coping strategies among caregivers of patients with schizophrenia in Malaysia. These findings can assist respective stakeholders in designing and expanding current intervention strategies in clinical practice.

## Methods

A cross-sectional study was conducted to investigate the use of coping strategies and their relationship to socio-demographic characteristics, psychological distress, and social support. This research focused on the caregivers of patients with schizophrenia who had attended the psychiatry clinic at Hospital Tengku Ampuan Afzan, Kuantan, in the span of 4 months from December 1^st^ 2021 to March 31^st^ 2022. The convenience sampling method was used to include caregivers who are 18 years or older, able to read and speak English or Malay proficiently, and able to provide informed written consent. A caregiver is someone who has spent at least 1 year taking care of the daily needs of the patient, such as ensuring the patient takes medication on time, taking the patient to hospital appointments, staying with the patient if the patient is hospitalized, and managing overall communication with the hospital team regarding the patient's illness (5). Caregivers with <1 year of experience and those who had a psychiatric diagnosis prior to the study are excluded from the study.

The sample size calculation was based on the Lwanga and Lemeshow scale by using the following formula: *n* = [z/d]2 p (1-p), where *n* = sample size, *p* = expected prevalence, *z* = 1.96, a 95% level of confidence, and *d* = 5% precision. In Malaysia, it is reported that 31.5% of caregivers of schizophrenia patients have suffered psychological distress (2). As a result, *p* = 31.5%. Based on the Lwanga and Lemeshow scale and by using the formula, the estimated sample size required was 331 patients.

The caregivers who had attended the psychiatric clinic in HTAA every morning from Monday to Friday were screened according to the inclusion and exclusion criteria. Those who met the inclusion criteria were given a brief explanation of the study as well as a participant information sheet that they could keep and refer to. The consent form was distributed to the willing caregivers. Those who did not consent will be excluded from the study. To maintain privacy, consenting caregivers were taken to a designated room. Demographic data was gathered, and caregivers were given a set of questionnaires written in Malay. The data for this study was gathered by a well-trained researcher who was fluent in Malay and was aware of the participants' social and cultural backgrounds. The clinical information about the patient being cared for was gathered from caregivers through self-report. Participants were given 15 min to complete the questionnaire. If the level of psychological distress was very high and if the caregivers agreed, a personal consultation would be provided and they would be referred to the psychiatric clinic.

The socio-demographic information obtained included the age, gender, ethnicity, relationship with the patient, employment status, types of employment, monthly income (in Ringgit Malaysia or RM), education level, duration of caregiving (in years), and clinical data. There are three different income groups in Malaysia based on the national socioeconomic status (9), namely: the top 20% (T20) or income of RM 10,960 and above; the middle 40% (M40) or income ranging from RM 4,850 to RM 10,959; and the bottom 40% (B40) or income of RM 4,849 and below. The exchange rate at the time of data collection was RM 4.45 to USD 1.00. Clinical data included the patient's illness duration (in years), onset age (in years), number of hospitalizations, patient's employment status, and number of admissions.

The Brief-COPE, which is designed to assess respondents' coping skills, consists of 28 items that are assessed on a four-point Likert scale (from 1 to 4, with 1 indicating “I haven't done this at all” and 4 indicating “I have done this a lot”). There are 14 domains on the scale, with two categories in each domain. The domains are further divided into three coping groups: emotion-focused coping (humor, religion, acceptance, and emotional support); problem-focused coping (instrumental support, active coping, positive reframing, and planning); and avoidance coping (self-distraction, venting, self-blame, behavioral disengagement, denial, and substance use) (14, 19). The higher the score, the more coping methods were applied (20). The Malaysian Brief-COPE version is an effective and reliable instrument for analyzing Malaysian respondents. The Malaysian population's internal consistency on Brief-COPE was 0.83 (21).

To assess psychological distress, the Kessler 10 (K10) was utilized. It has been adopted across the world where English is spoken as a screening and outcome measure in mental health surveys and primary care settings, where it assesses and analyses serious mental illness and nonspecific psychological distress (22). The Kessler 10 and Kessler 6 used in Malaysia are valid and reliable to assess people suffering from nonspecific psychological distress (23). The reliability value of the K10 scale for one study involving caregivers of patients with schizophrenia was 0.87 (2). The range of the scale from 10 to 19 shows no signs of distress; the range from 20 to 24 shows mild signs of distress; the range from 25 to 29 shows a moderate amount of distress; and the range from 30 to 50 shows that the person is in a lot of distress (2).

Furthermore, the Multidimensional Scale of Perceived Social Support (MSPSS) is a questionnaire tool with a 12-item scale measure and each item is subdivided into four categories. It is a famous tool, known to be reliable, fair, valid, and strong in scale of measure (24). It has been validated among Malaysians and translated into the Malay language (25). The MSPSS has good internal consistency with a Cronbach's alpha value of 0.86 (26).

The data retrieved from the above-mentioned tools was then analyzed using SPSS (Statistical Package for the Social Sciences) 27. The descriptive analysis was carried out to describe and summarize the variables in the study. The coping strategies are the dependent variables in this study, while the independent variables are the age, gender, ethnicity, relationship with the patient, employment status, types of employment, monthly income, education level, caregiving duration (in years), patient's illness duration (in years), onset age (in years), number of hospitalizations, patient's employment status, and number of admissions. The psychological distress, perceived social support, and coping strategies scores are treated as numerical variables. Continuous variables, on the other hand, were given as standard deviation and mean, and finally, frequency and percentage represented the categorical data. On continuous data, a normality test was performed using the Kolmogorov Smirnov and graphical methods. The distribution of samples was found to be of normal distribution. In this study, analysis of variance (ANOVA) and independent *t*-test were utilized to investigate the differential attributes of the variables. Simple linear regression was used to further analyze the results and identify the unadjusted relationship between the independent variables and the research findings. Any variable with a *p* < 0.05 is regarded as significant and was selected for multiple linear regression. Subsequently, multivariate linear regressions were used to determine the potential predictors associated with coping strategies.

This study was approved by the UiTM Research Ethics Committee (REC/06/2021 (MR/384) and the National Medical Research and Ethics Committee (MREC) Ministry of Health Malaysia through the National Medical Research Registry (NMRR) (Registration number: NMRR ID-22-00016-BH8).

## Results

A total of 334 respondents were recruited for this study. However, only 331 of them completed the questionnaire, with a 99.1% response rate. The mean age of the respondents is 51 (SD = 16), and the majority of them are Malay (*n* = 272, 82.8%); married (*n* = 215, 65%); have a secondary level of education (*n* = 184, 55.6%); currently employed (*n* = 176, 53.2%); and come from low economic status with a household monthly income of RM 4,849 per month and below (*n* = 286, 86.4%).

The majority of participants (*n* = 115, 34.7%) are mothers of patients with schizophrenia, with a mean caretaking duration of 9.34 years (SD = 8.58). The majority of patients receiving care are unemployed (*n* = 274, 82.8%), with a mean illness duration of 11.94 years (SD = 9.70), an onset age of 27.59 years (SD = 9.15), and 1.92 years (SD = 2.27) of previous admissions to the psychiatric ward.

The majority of respondents (*n* = 210, 63.4%) reported receiving moderate social support. However, 15.7 % of respondents (*n* = 52) were experiencing psychological distress, with a mean and standard deviation of 14.95 and 5.73, respectively. The group's most common coping strategy was emotion-focused coping (mean = 5.42, SD = 1.18), followed by problem-focused coping and avoidance coping (refer to Table 1).

**Table 1:** Sociodemographic characteristics of caregivers of patients with schizophrenia (*n* = 331).

**Factors**	***n*** **(%)**	**Mean (SD)**
**Age**		50.92 (16.22)
**Gender**		
Male	128 (38.7)	
Female	203 (61.3)	
**Ethnicity**		
Malay	272 (82.2)	
Chinese	44 (13.3)	
Indian	9 (2.7)	
Other	6(1.8)	
**Marital status**		
Single	57 (17.2)	
Married	215 (65)	
Divorced	59 (17.8)	
**Employment status**		
Employed	176 (53.2)	
Unemployed	155 (46.8)	
**Total household income**		
RM 4,849/month and below	286 (86.4)	
RM 4,850–RM 10,959/month	40 (12.1)	
RM 10,960 and above/month	5 (1.5)	
**Education level**		
No formal education	14 (4.2)	
Primary	60 (18.1)	
Secondary	184 (55.6)	
Tertiary	73 (22.1)	
**Relationship with patient with Schizophrenia**		
Father	37 (11.2)	
Mother	115 (34.7)	
Spouse	40 (12.1)	
Siblings	77 (23.3)	
Children	30 (9.1)	
Other	32 (9.7)	
**Duration of caregiving (in years)**		9.34 (8.58)
**Psychological distress**		14.95 (5.73)
No distress	279 (84.3)	
Mild distress	31 (9.4)	
Moderate distress	12 (3.6)	
Severe distress	9 (2.7)	
**Perceived social support**		60.12 (12.11)
Low support	39 (11.8)	
Moderate support	210 (63.4)	
High support	82 (24.8)	
**Coping strategies**		
Problem-focused coping		5.02 (1.56)
Emotion-focused coping		5.43 (1.18)
Avoidance coping		3.19 (0.87)
**Patient receiving care/support**		
**Age of onset (in years)**		27.59 (9.15)
**Duration of illness (in years)**		11.94 (9.70)
**Number of previous hospitalizations**		1.92 (2.27)
**Patient's employment status**		
Employed	57 (17.2)	
Unemployed	274(82.8)	

Table 2 shows the standard deviation and mean scores of the caregivers' 14 coping mechanisms. Positive reframing (SD = 1.68, mean = 5.97), religion (SD = 1.4, mean = 6.92), and acceptance (SD = 1.67, mean = 5.87) were the top three coping strategies. The findings revealed that religious coping is the most commonly used method of coping, while substance use is the least commonly used method of coping (SD = 0.27, mean = 2.03).

**Table 2:** Mean score and standard deviation of coping strategies among caregivers of patients with schizophrenia.

**Coping strategies**	**Mean (SD)**
**Problem-focused**	
Active coping	5.34 (1.82)
Planning	5.29 (1.79)
Instrumental support	4.44 (1.91)
Positive reframing	5.97 (1.68)
**Emotion-focused**	
Emotional support	4.30 (1.93)
Acceptance	5.87 (1.67)
Religion	6.92 (1.50)
Humor	4.07 (1.37)
**Avoidance**	
Self-distraction	5.18 (1.65)
Venting	3.58 (1.66)
Denial	3.00 (1.44)
Behavioral disengagement	2.58 (1.25)
Substance use	2.03 (0.27)
Self-Blame	2.76 (1.22)

Table 3 shows the relationship between psychological distress, socio-demographic characteristics, perceived social support, and three major coping strategies in caregivers. Generally, all coping dimensions were found to be significantly linked to the respondents' educational level and ethnicity (*p* < 0.001). Gender was only significantly associated with an emotion-focused coping style, whereas employment status was only significantly associated with a problem-focused coping style. Marital status and certain types of relationships were both significantly associated with emotion-focused and avoidance coping styles.

**Table 3:** Factors associated with coping strategies among caregivers of patients with schizophrenia.

**Factors**		**Problem-focused coping**	**Emotion-focused coping**	**Avoidance coping**
**Caregivers**				
Age		−0.351^c^ (<0.001)^***^	−0.187^c^ (<0.001)^***^	−0.326^c^ (<0.001)^***^
Gender	Male	−0.100^a^ (0.921)	−2.524^a^ (0.012)^*^	−1.242^a^ (0.215)
	Female			
Ethnicity	Malay	10.158^b^ (<0.001)^***^	23.673^b^ (<0.001)^***^	8.360^b^ (<0.001)^***^
	Chinese			
	Indian			
	Other			
Marital status	Single	5.781^b^ (0.003)^**^	1.160^b^ (0.315)	5.696^b^ (0.004)^**^
	Married			
	Divorced			
Employment status	Employed	3.381^a^ (<0.001)^***^	0.750^a^ (0.454)	1.226^a^ (0.221)
	Unemployed			
				
Education level	No formal education	22.531^b^ (<0.001)^***^	15.776^b^ (<0.001)^***^	9.825^b^ (<0.001)^***^
	Primary			
	Secondary			
	Tertiary			
				
Relationship with patient	Father	3.287^b^ (0.007)^**^	1.062^b^ (0.382)	3.266^b^ (0.007)^**^
	Mother			
	Sibling			
	Spouse			
	Children			
	Other			
Total household monthly income		0.138^c^ (0.010)^*^	0.170^c^ (0.002)^**^	0.112^c^ (0.042)^*^
Duration of caregiving (in years)		−0.278^c^ (<0.001)^***^	−0.238^c^ (<0.001)^***^	−0.251^c^ (<0.001)^***^
Psychological distress		0.159^c^(0.004)^**^	0.166^c^(0.002)^**^	0.438^c^(<0.001)^***^
Perceived social support		0.350^c^(<0.001) ^***^	0.336^c^(<0.001) ^***^	0.072^c^(0.190)
**Patient receiving care/support**				
Age of onset (in years)		0.052^c^ (0.350)	0.073^c^ (0.188)	0.106^c^ (0.055)
Duration of illness (in years)		−0.142^c^ (0.01)^*^	−0.175^c^ (0.001)^**^	−0.159^c^ (0.004)^**^
Number of previous hospitalizations		−0.096^c^ (0.081)	−0.090^c^ (0.103)	−0.048^c^ (0.386)
Patient's employment status	Employed	0.605^a^ (0.546)	−0.035^a^ (0.972)	0.508^a^ (0.612)
	Unemployed			

a t-Independent t-test,

b F-ANOVA,

c Pearson correlation coefficient,

* significance at p < 0.05,

** significance at p < 0.01,

*** significance at p < 0.001.

The research analysis illustrates that perceived social support, duration of disease and caregiving, household income, and psychological distress are considerably related to every coping strategy. Psychological distress and perceived social support, in particular, are positively associated with the emotion-focused coping strategy (coefficients of 0.336 and 0.166, respectively) and the problem-focused coping style (coefficients of 0.35 and 0.159, respectively). Furthermore, it was observed that in terms of avoidance coping strategy, psychological distress and perceived social support are positively associated (coefficient 0.438 and coefficient 0.072, respectively). In the meantime, the illness period, the caregiving period, and the caregivers' age are negatively correlated with the emotion-focused, problem-focused, and avoidance coping mechanisms. However, there was no statistically significant association between the onset age and the number of previous hospitalizations for each coping dimension.

The predictors of coping strategies were determined using multiple regression analysis. The normality assumption was not violated. In consideration of the multicollinearity where various variables are correlated, all clinical and demographic factors were combined into a linear regression model. However, it is seen that with high tolerance for all variables, multicollinearity is not present in the regression model. Analysis was conducted independently for each of the coping groups. Univariate linear regression was conducted, followed by multivariate linear regression.

There were significant differences in the use of problem-focused coping strategies by Chinese vs. Malays (β = −2.169, *p*-value = 0.001), those with secondary education versus those without (β = 2.901, *p*-value = 0.009), and those with tertiary education vs. those without (β = 4.579, *p*-value < 0.001). There were also positive associations between problem-focused coping strategies and perceived social support (β = 0.107, *p*-value < 0.001) and psychological distress (β = 0.151, *p*-value < 0.001). Table 4 shows that participants' scores on psychological distress, perceived social support, ethnicity, and education level made a statistically significant contribution to the increased score in problem-focused coping strategies and explains 36.5% of the variation in the model (*r*^**2**^ = 0.365, *F* = 12.046, *p* < 0.001).

**Table 4:** Factors associated with problem focus coping strategies among caregivers of patients with schizophrenia.

**Factors**	**Simple linear regression**				**Multiple linear regression**			
	**β**	**95%CI**		***p*** **-value**	**β**	**95%CI**		***p*** **-value**
**Constant**					5.355			
**Caregivers**								
**Age**	−0.101	−0.130	−0.072	<0.001	−0.018	−0.066	0.029	0.448
**Gender**								
Female	Ref	Ref	Ref	Ref				
Male	−0.052	−1.089	0.984	0.921				
**Ethnicity**								
Malay	Ref	Ref	Ref	Ref	Ref	Ref	Ref	Ref
Chinese	−3.896	−5.328	−2.465	<0.001	−2.169	−3.479	−0.859	0.001
Indian	−1.336	−4.320	1.648	0.379	−0.373	−3.068	2.321	0.785
Other	−2.836	−6.471	0.799	0.126	−2.090	−5.344	1.165	0.207
**Marital status**								
Single	Ref	Ref	Ref	Ref	Ref	Ref	Ref	Ref
Married	−1.649	−2.995	−0.303	0.017	−0.175	−1.546	1.196	0.802
Divorced	−1.649	−4.565	−1.208	<0.001	−0.295	−2.089	1.500	0.747
**Employment status**								
Employed	Ref	Ref	Ref	Ref	Ref	Ref	Ref	Ref
Unemployed	−1.709	−2.703	−0.715	<0.001	−0.456	−1.431	0.519	0.358
**Education level**								
No formal education	Ref	Ref	Ref	Ref	Ref	Ref	Ref	Ref
Primary	1.779	−0.710	4.267	0.161	1.131	−1.145	3.406	0.329
Secondary	4.684	2.360	7.008	<0.001	2.901	0.731	5.071	0.009
Tertiary	7.100	4.654	9.546	<0.001	4.579	2.188	6.970	<0.001
**Relationship with patient**								
Father	Ref	Ref	Ref	Ref	Ref	Ref	Ref	Ref
Mother	0.337	−1.366	2.040	0.697	0.189	−1.352	1.730	0.809
Spouse	1.014	−1.041	3.070	0.332	0.426	−1.502	2.354	0.664
Siblings	2.332	0.529	4.135	0.011	0.818	−1.051	2.686	0.390
Children	2.456	0.242	4.670	0.030	0.288	−2.214	2.790	0.821
Other	2.564	0.389	4.740	0.021	0.430	−1.834	2.694	0.709
**Total household monthly income**								
RM 4,849/month and below	Ref	Ref	Ref	Ref	Ref	Ref	Ref	Ref
RM 4,850-RM 10,959/month	1.864	0.326	3.401	0.018	0.021	−1.411	1.454	0.977
RM 10,960 and above/month	1.789	−2.320	5.898	0.392	−0.573	−4.189	3.042	0.755
**Duration of caregiving (in years)**	−0.151	−0.208	−0.094	<0.001	−0.081	−0.169	0.006	0.067
**Psychological distress**	0.129	0.042	0.216	0.004	0.151	0.074	0.228	<0.001
**Perceived Social support**	0.135	0.096	0.174	<0.001	0.107	0.070	0.144	<0.001
**Patient receiving care/support**								
**Age of onset (in years)**	0.026	−0.029	0.081	0.350				
**Duration of caregiving (in years)**	−0.068	−0.120	−0.017	0.010	0.019	−0.056	0.093	0.622
**Patient's employment status**								
Employed	Ref	Ref	Ref	Ref				
Unemployed	−0.411	−1.747	0.925	0.546				
**Number of previous hospitalizations**	−0.197	−0.417	0.024	0.081				

*r*^**2**^ = 0.365, *F* = 12.046, *p* < 0.001.

The participants' scores on psychological distress, perceived social support, ethnicity, and education level also made a statistically significant contribution to the increased score in emotion-focus coping strategies and explained 36.8% of the variation in the model (*r*^**2**^ = 0.368, *F* = 13.131, *p* < 0.001) as shown in Table 5. Additionally, there were significant differences in the use of coping strategies by Chinese vs. Malays (β = −5.026, *p*-value < 0.001), those with secondary education vs. those without (β = 3.254, *p*-value = 0.018), and those with tertiary education vs. those without (β = 5.867, *p*-value < 0.001). Emotion-focused coping strategies were also associated with perceived social support (β = 0.124, *p*-value < 0.001) and psychological distress (β = 0.195, *p*-value < 0.001).

**Table 5:** Factors associated with emotion focus coping strategies among caregivers of patients with schizophrenia.

**Factors**	**Simple linear regression**				**Multiple linear regression**			
	**β**	**95%CI**		***p*** **-value**	**β**	**95%CI**		***p*** **-value**
**Constant**					14.561			
**Caregivers**								
**Age**	−0.068	−0.107	−0.029	<0.001	0.012	−0.028	0.053	0.544
**Gender**								
Female	Ref	Ref	Ref	Ref	Ref	Ref	Ref	Ref
Male	−1.664	−2.961	−0.367	0.012	−0.857	−1.953	0.238	0.124
**Ethnicity**								
Malay	Ref	Ref	Ref	Ref	Ref	Ref	Ref	Ref
Chinese	−7.015	−8.729	−5.301	<0.001	−5.026	−6.646	−3.405	<0.001
Indian	−4.042	−7.616	−0.469	0.027	−2.340	−5.628	−0.948	0.162
Other	−5.098	−9.452	−0.744	0.022	−3.630	−7.638	−0.378	0.076
**Marital status**								
Single	Ref	Ref	Ref	Ref				
Married	−0.911	−2.636	0.814	0.300				
Divorced	−1.662	−3.812	0.489	0.129				
**Employment status**								
Employed	Ref	Ref	Ref	Ref				
Unemployed	−0.487	−1.764	0.790	0.454				
**Education level**								
No formal education	Ref	Ref	Ref	Ref	Ref	Ref	Ref	Ref
Primary	2.564	−0.664	5.793	0.119	1.589	−1.232	4.410	0.269
Secondary	4.644	1.628	7.659	0.003	3.254	0.573	5.935	0.018
Tertiary	8.153	4.979	11.326	<0.001	5.867	2.930	8.804	<0.001
**Relationship with patient**								
Father	Ref	Ref	Ref	Ref				
Mother	0.912	−1.276	3.101	0.413				
Spouse	−0.370	−3.011	2.271	0.783				
Siblings	1.613	−0.703	3.929	0.172				
Children	0.230	−2.615	3.075	0.874				
Other	1.980	−0.816	4.775	0.164				
**Total household monthly income**								
RM 4,849/month and below	Ref	Ref	Ref	Ref	Ref	Ref	Ref	Ref
RM 4,850-RM 10,959/month	2.542	0.602	4.481	0.010	0.256	−1.470	1.981	0.771
RM 10,960 and above/month	2.617	−2.567	7.800	0.321	−0.239	−4.616	4.139	0.915
**Duration of caregiving (in years)**	−0.164	−0.236	−0.091	<0.001	−0.058	−0.159	0.042	0.255
**Psychological distress**	0.171	0.061	0.281	0.002	0.195	0.100	0.290	<0.001
**Perceived Social support**	0.164	0.114	0.213	<0.001	0.124	0.078	0.170	<0.001
								
**Patient receiving care/support**								
**Age of onset (in years)**	0.047	−0.023	0.116	0.188				
**Duration of illness (in years)**	−0.106	−0.171	−0.041	0.001	−0.015	−0.095	0.066	0.717
**Patient's employment status**								
Employed	Ref	Ref	Ref	Ref				
Unemployed	0.030	−1.659	1.719	0.972				
**Number of previous hospitalizations**	−0.232	−0.512	0.047	0.103				

r^**2**^ = 0.368, F = 13.131, p < 0.001.

Moreover, the participants' scores on psychological distress, education level, and ethnicity made a statistically significant contribution to the increased score in avoidance coping strategies and explained 36.1% of the variation in the model (*r*^**2**^ = 0.361, *F* = 10.409, *p* < 0.001). Table 6 shows that there were significant differences in the use of avoidance coping strategies by Chinese vs. Malays (β = −2.478, *p*-value < 0.001) and those with tertiary education vs. those without (β = 3.390, *p*-value = 0.010). There was also a positive association between avoidance coping strategies and psychological distress (β = 0.376, *p*-value < 0.001).

**Table 6:** Factors associated with avoidance coping strategies among caregivers of patients with schizophrenia.

**Factors**	**Simple linear regression**				**Multiple linear regression**			
	**β**	**95%CI**		***p*** **-value**	**β**	**95%CI**		***p*** **-value**
**Constant**					13.569			
**Caregivers**								
**Age**	−0.105	−0.138	−0.138	<0.001	−0.037	−0.088	0.015	0.160
**Gender**								
Female	Ref	Ref	Ref	Ref				
Male	−0.731	−1.889	0.427	0.215				
**Ethnicity**								
Malay	Ref	Ref	Ref	Ref	Ref	Ref	Ref	Ref
Chinese	−4.100	−5.716	−2.485	<0.001	−2.478	−3.928	−1.029	<0.001
Indian	0.087	−3.282	3.455	0.960	−1.128	−4.102	1.845	0.456
Other	−0.691	−4.795	3.412	0.741	−1.843	−5.390	1.704	0.307
**Marital status**								
Single	Ref	Ref	Ref	Ref	Ref	Ref	Ref	Ref
Married	−1.798	−3.307	−0.290	0.020	−0.023	−1.532	1.485	0.976
Divorced	−3.216	−5.097	−1.335	0.001	−1.168	−3.159	0.822	0.249
**Employment status**								
Employed	Ref	Ref	Ref	Ref				
Unemployed	−0.705	−1.835	0.426	0.221				
**Education level**								
No formal education	Ref	Ref	Ref	Ref	Ref	Ref	Ref	Ref
Primary	0.557	−2.376	3.490	0.709	0.452	−2.085	2.989	0.726
Secondary	3.286	0.547	6.026	0.019	1.909	−0.496	4.315	0.119
Tertiary	4.830	1.947	7.713	0.001	3.390	0.806	5.975	0.010
**Relationship with patient**								
Father	Ref	Ref	Ref	Ref	Ref	Ref	Ref	Ref
Mother	2.185	0.277	4.094	0.025	1.171	−0.503	2.845	0.170
Spouse	1.674	−0.629	3.978	0.154	0.629	−1.516	2.774	0.5644
Siblings	3.675	1.655	5.695	<0.001	1.585	−0.471	3.641	0.130
Children	3.458	0.977	5.939	0.006	0.770	−2.004	3.544	0.585
Other	3.418	0.980	5.856	0.006	0.596	−1.920	3.111	0.641
**Total household monthly income**								
RM 4,849/month and below	Ref	Ref	Ref	Ref				
RM 4,850–RM 10,959/month	1.317	−0.415	3.049	0.136				
RM 10,960 and above/month	1.242	−3.388	5.871	0.598				
**Duration of caregiving (in years)**	−0.152	−0.216	−0.089	<0.001	−0.048	−0.145	0.049	0.329
**Psychological distress**	0.399	0.310	0.488	<0.001	0.376	0.292	0.461	<0.001
**Perceived Social support**	0.031	−0.015	0.078	0.190				
**Patient receiving care/support**								
**Age of onset (in years)**	0.060	−0.001	0.122	0.055				
**Duration of illness (in years)**	−0.086	−0.143	−0.028	0.004	0.001	−0.082	0.085	0.973
**Patient's employment status**								
Employed	Ref	Ref	Ref	Ref				
Unemployed	−0.387	−1.883	1.110	0.612				
**Number of previous hospitalizations**	−0.110	−0.358	0.139	0.386				

r^**2**^ = 0.361, F = 10.409, p < 0.001.

## Discussion

This study attempted to focus on the limitations of the literature in adopting effective coping strategies by care takers of schizophrenia patients by examining perceived social support, psychological distress, and socio-demographic factors. According to the acknowledged information, this is the first Malaysian research to address the association between coping mechanisms, perceived social support, and psychological distress among caregivers of schizophrenia patients, who employ both effective and ineffective coping mechanisms to deal with the challenges they face (6). The notion of coping differs among caregivers (7), thus it is difficult to compare the outcomes of various research due to variances in the assessment scales utilized in each (12). This study demonstrated that caregivers prefer using emotion-focused coping strategies more, which is also in line with other research (27, 28).

The findings of this study also revealed that religious coping is the most commonly used method of coping. Religion is widely believed to be the most prevalent coping strategy employed by caregivers of patients diagnosed with schizophrenia. It is hypothesized to be an effective method of coping with the situation, regardless of the religious belief, its manifestation, or the location (religious institution or home) (21). Moreover, it has been discovered that a religious coping mechanism can mitigate the impact of depression-related identity loss (29). In other words, spiritual belief promotes health and serves as a source of optimism (30). Therefore, it is observed that a patient with a religious coping strategy is more capable of handling and surviving more severe life challenges (21). In patient-involved research, it has been found that patients with increased symptoms and poor functioning are more likely to read the Bible or pray as a coping mechanism (18). To deal with a patient's aggressive behavior, the caregiver engages in their work, accepts their aggression, accepts assistance from others, and prays (31). Positive religious coping is when an individual asks God for help, involves God in every difficult situation, and surrenders to God when life problems become overwhelming (12).

In comparison to other studies, substance use was the least utilized coping strategy (mean = 2.03, SD = 0.27) in the present study (32, 33). This may be due to the fact that 60% of the Malaysian population is Muslim (29). Similar to alcohol, an illegal drug used for recreational purposes is prohibited in Islam, regardless of the quantity, because it can be addictive and contribute to negative outcomes (29, 30). Furthermore, for alcohol consumption, Malaysia is among the most successful nations in reducing harm and decreasing alcohol consumption per capita between 2010 and 2017 as a result of its effective tax methods and alcohol control policies (31). Sixty-four percentage of Malaysian youth in a separate study agreed that the country should be concerned about alcohol-related issues (34).

The results of this study also indicate that female caregivers engage in significantly more emotion-focused coping strategies than their male counterparts. Additionally, women sought social support at a greater rate than men (40). Their primary coping strategy is the search for support, which includes expressing emotions such as crying, disclosing feelings to reduce stress, and participating in group support activities, which are linked to their religiosity (13).

When included in multiple linear regression, only ethnicity and education level are significantly associated with all three coping strategies. The tertiary level of education is associated with all three coping strategies, while the secondary education level was linked to emotion and problem-focused coping. The previous study's regression model illustrated that adaptive coping approaches were considerably related to education level (16). In terms of education, caregivers with a university education utilize considerably more problem-focused engagement strategies than caregivers with less education (27). This is further demonstrated by another study, which found that caregivers with considerably more abilities and knowledge are likely to use positive coping styles (35). The present study also revealed a significant association between the coping strategies and the Chinese community in the sample population. One study in China revealed that Chinese culture emphasizes the value of family, which plays a larger role in coping with stress when caring for patients with schizophrenia (22).

Other than that, the study observed that 15.7% of caregivers experienced psychological distress, which was consistent with other similar Malaysian research, which reported that 14% of the caregivers suffered from psychological distress (36). Intriguingly, 84.3% of respondents reported no caregiving-related distress, which suggests the need for additional research, particularly qualitative studies investigating how coping strategies can and have aided in their management of psychological distress.

Furthermore, family members of schizophrenia patients experience moderate stress and have few coping strategies (37). In this study, coping strategies were also found to have a significant association with psychological distress. This was consistent with other studies that revealed a significant positive correlation between the level of stress and coping status (38, 39). In our study, all three coping strategy groups (avoidance, emotion-focused, and problem-focused coping strategies) were also found to have significant associations with psychological distress. One study in Malaysia showed adaptive coping is linked to lower levels of psychological distress and morbidity in caregivers, as well as better patient outcomes (5). Other coping mechanisms that are linked with the caregiver's distress include self-blame and avoidance (32, 33). On the other hand, caregivers who experience less distress are linked with the following coping strategies: using religion, acceptance, active coping, positive reframing, and seeking emotional support (32).

Moreover, the high percentage of good social support in this study may point to the positive culture and nature of Asian society. The caregivers of patients diagnosed with schizophrenia may cope better with the difficulties if they are provided with social support to strengthen their psychological resilience (29). Consistent with another study that showed a link between coping and social support (39), we also found a significant association between emotional and problem-focused coping and perceived social support. However, it failed to show a significant association with avoidance coping. In other studies (30), the levels of social support and coping strategies were shown to have a low negative correlation (*r* = −0.195 and *p* = 0.048). Poor social support has been linked to the adoption of emotion-focused coping techniques, whereas problem-focused coping strategies have been linked to higher levels of practical and emotional social support, as well as professional assistance (31). Finally, coping styles that are associated with less social support include the utilization of religious help and resignation (34).

The present study has some potential limitations. The first factor to be noted is that it is not feasible to determine the direction of the relationship between complex factors of the coping strategies because it is a cross-sectional study. Second, the sample was comprised predominantly of caregivers of patients diagnosed with schizophrenia in outpatient care, and there was a lack of inclusion of other psychiatric settings. Third, due to the multifactorial nature of coping strategies, important factors that may affect the outcome of coping or distress, such as the current or most recent episodes of schizophrenia, either in remission or relapse, the severity of symptoms, and social function, were not assessed in the study. Given that the factors examined together in the regression analyses only contributed approximately 36–37% of the variance in coping strategy utilization, other factors may be contributing to variance in coping strategy utilization among caregivers, such as religiosity, caregiver burden, patient psychopathology, and quality of life. Lastly, the data were assessed using a variety of self-report measures. Therefore, the relationships between constructs may be impacted by shared method variance as well as social desirability and other response biases. This is especially relevant considering the gender differences in reported coping strategies as well as attitudes and beliefs about substance use in Malaysia. As such, these findings should be interpreted with caution.

## Conclusion

The present study revealed a correlation between psychological distress, perceived social support, and coping strategies. Psychological distress, along with other factors such as ethnicity and level of education, has been linked to coping mechanisms. Therefore, the physician should assist caregivers in maintaining a healthy coping style and steer them away from maladaptive coping styles, such as substance abuse. Both the presence of maladaptive coping strategies and the absence of adaptive coping strategies contribute to the negative effects of mental illness.

## Data availability statement

The datasets presented in this article are not readily available because privacy concerns. Requests to access the datasets should be directed to drtgsaifuddin@gmail.com.

## Ethics statement

The studies involving human participants were reviewed and approved by UiTM Research Ethics Committee (REC/06/2021 (MR/384) and the National Medical Research and Ethics Committee (MREC) Ministry of Health Malaysia *via* the National Medical Research Registry (NMRR) (Registration number: NMRR ID-22-00016-BH8). The patients/participants provided their written informed consent to participate in this study.

## Author contributions

SA, TKa, and AN contributed to the conception and design of the study. TKh organized the database and wrote the first draft of the manuscript. NA performed the statistical analysis. SA, TKa, AN, and NA wrote sections of the manuscript. All authors contributed to manuscript revision, read, and approved the submitted version.

## Conflict of interest

The authors declare that the research was conducted in the absence of any commercial or financial relationships that could be construed as a potential conflict of interest.

## Publisher's note

All claims expressed in this article are solely those of the authors and do not necessarily represent those of their affiliated organizations, or those of the publisher, the editors and the reviewers. Any product that may be evaluated in this article, or claim that may be made by its manufacturer, is not guaranteed or endorsed by the publisher.
